# Supplementary material for: Taotie neurons regulate appetite in Drosophila
Source: Nat Commun. 2016 Dec 7;7:13633. doi: 10.1038/ncomms13633 (PMC5151092; doi:10.1038/ncomms13633)
Supplement: Supplementary Information — Supplementary Figures 1-20, Supplementary Table 1. [file ncomms13633-s1.pdf]

Supplementary Figure 1. PER and food-intake of genetic controls during activation and inactivation of Taotie neurons.

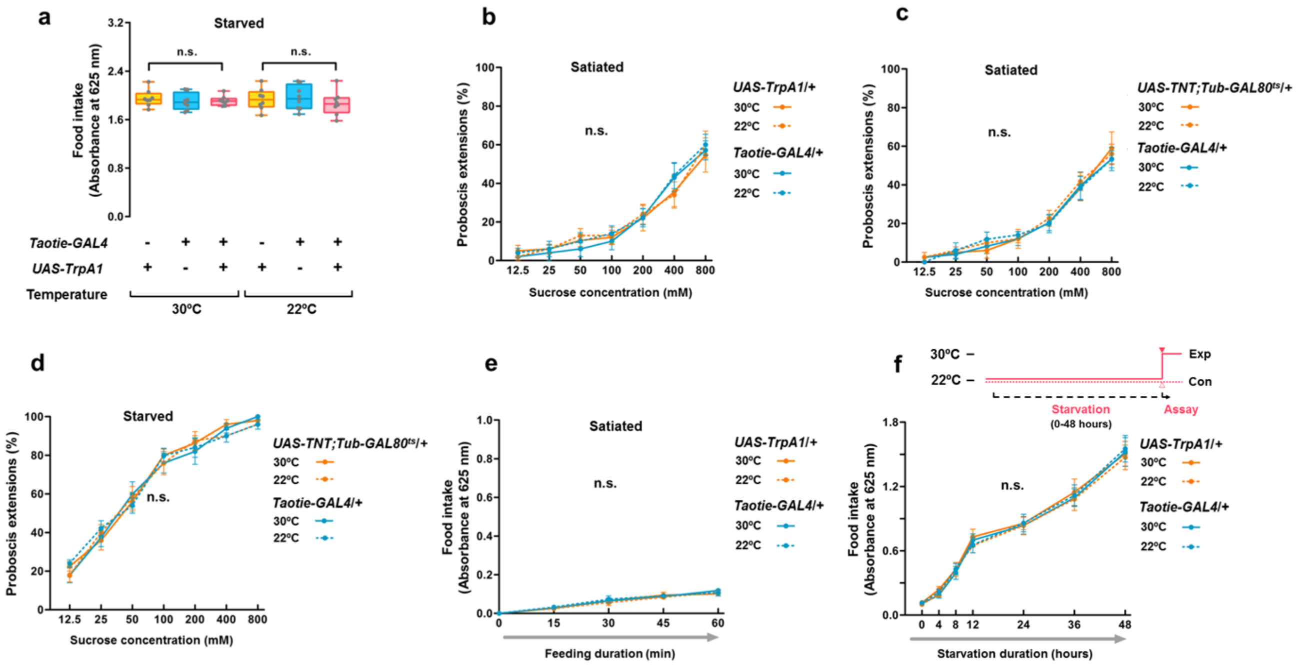

(a) Colorimetric quantification of food-intake in starved *Taotie-GAL4*/*UAS-TrpA1* flies. For starvation treatment, *Taotie-GAL4*/*UAS-TrpA1* and genetic control flies were starved 60-64 hours at 22ºC before being subjected to behavioral tests. N = 8 groups per condition, n = 20 flies in each group. (b) Fractions of satiated flies (*Taotie-GAL4*/+ and *UAS-TrpA1*/+) showing PER to the concentrations of sucrose at 30ºC and 22ºC (N = 4-5, n = 5-13). (c,d) Fraction of *Taotie-GAL4*/+ flies and *UAS-TNT;Tub-GAL80^{ts}*/+ flies showing PER to different concentrations of sucrose in satiated (c) and starved (d) conditions at 30ºC and 22ºC (N = 4-5, n = 8-11). (e) The quantities of food ingested in satiated *Taotie-GAL4*/+ flies and *UAS-TrpA1*/+ flies within 1 hour feeding assay at 30ºC and 22ºC (N = 8). (f) Food-intake of *Taotie-GAL4*/+ flies and *UAS-TrpA1*/+ flies after specified duration of starvation at 22ºC (N = 8). The feeding test was performed at 30ºC or 22ºC as indicated. All genotypes, temperatures, and experimental conditions were as indicated with the plots. In a box and whisker plot, whiskers mark minimum and maximum, box includes 25th to 75th percentile, and the line in box indicates median of the data set. n.s. indicates not significant (p > 0.05) (Student *t* test within each

genotype for two group-only comparisons, ANOVA with Bonferroni post hoc test for multiple comparisons). Error bars indicate s.e.m.

Supplementary Figure 2. Obese phenotypes caused by activation of Taotie neurons.

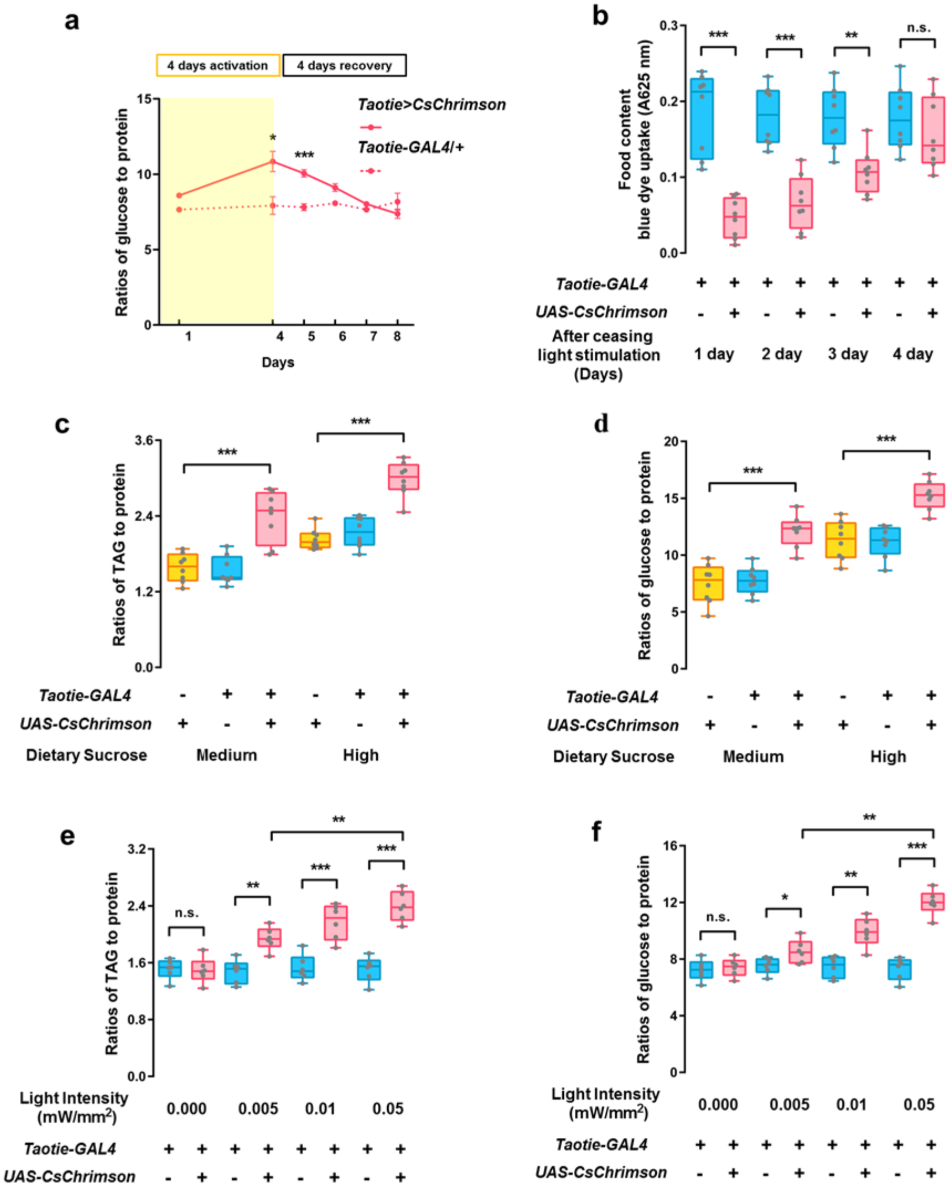

Taotie-GAL4/*UAS-CsChrimson* and genetic control flies were activated following continuous light exposure for 4 days, and then allowed to recover for another 4 days in the absence of light. Flies had *ad libitum* access to food during the entire time. The following tests were conducted at two time points: 1) after 4 days of activation, and 2) after 4 days of recovery. (a) Glucose levels, normalized to protein levels, in *Taotie-GAL4*/*UAS-CsChrimson* and genetic control flies (N = 6). (b) Food content in *Taotie-GAL4*/*UAS-CsChrimson* flies on different days after ceasing light stimulation (N = 8). (c)

Triglyceride levels, normalized to protein levels, in *Taotie-GAL4*/*UAS-CsChrimson* and genetic control flies on normal food and high sugar diet for 6 days (N = 8). (d) Glucose levels, normalized to protein levels, in *Taotie-GAL4*/*UAS-CsChrimson* and genetic control flies on normal food and high sugar diet for 6 days (N = 8). (e,f) Comparing the levels of triglyceride and glucose in *Taotie-GAL4*/*UAS-CsChrimson* flies and control flies with different light intensities for 6 days (N = 6). All genotypes, temperatures, and experimental conditions were as indicated with the plots. In a box and whisker plot, whiskers mark minimum and maximum, box includes 25th to 75th percentile, and the line in box indicates median of the data set. n.s. indicates not significant ($p > 0.05$); *: $p < 0.05$, **: $p < 0.01$, ***: $p < 0.001$ (Student *t* test within each genotype for two group-only comparisons, ANOVA with Bonferroni post hoc test for multiple comparisons). Error bars indicate s.e.m.

Supplementary Figure 3. Obesity induced by activation of Taotie neurons impairs insulin and leptin signals and elicits inflammatory response in flies.

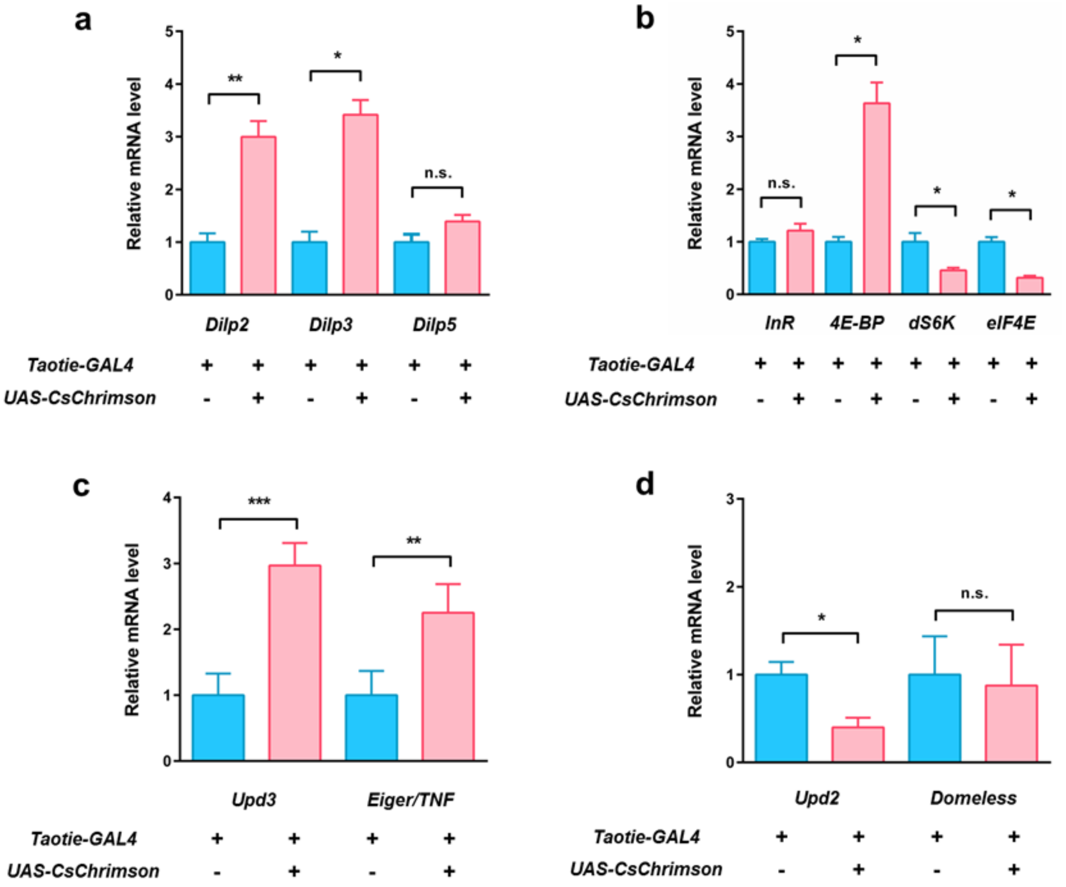

(a) Transcription levels of *Dilp2, Dilp3,* and *Dilp5* in *Taotie-GAL4*/*UAS-CsChrimson* and genetic control flies after 6 days of light stimulation. (b) Transcription levels of insulin receptor and related downstream regulators in *Taotie-GAL4*/*UAS-CsChrimson* and genetic control flies after 6 days on light stimulation. (c) Transcription levels of *Upd3* and *Eiger* in *Taotie-GAL4*/*UAS-CsChrimson* and genetic control flies after 6 days of light stimulation. (d) Transcription levels of *Upd2* and *Domeless* in *Taotie-GAL4*/*UAS-CsChrimson* and genetic control flies. All genotypes, temperatures, and experimental conditions are indicated with the plots. n.s. indicates not significant ($p > 0.05$); *: $p < 0.05$, **: $p < 0.01$, ***: $p < 0.001$ (Student's *t* test within each genotype for two group-only comparisons). Error bars indicate SEM.

Supplementary Figure 4. Behavioral responses of Taotie neurons and genetic control flies toward different food sources.

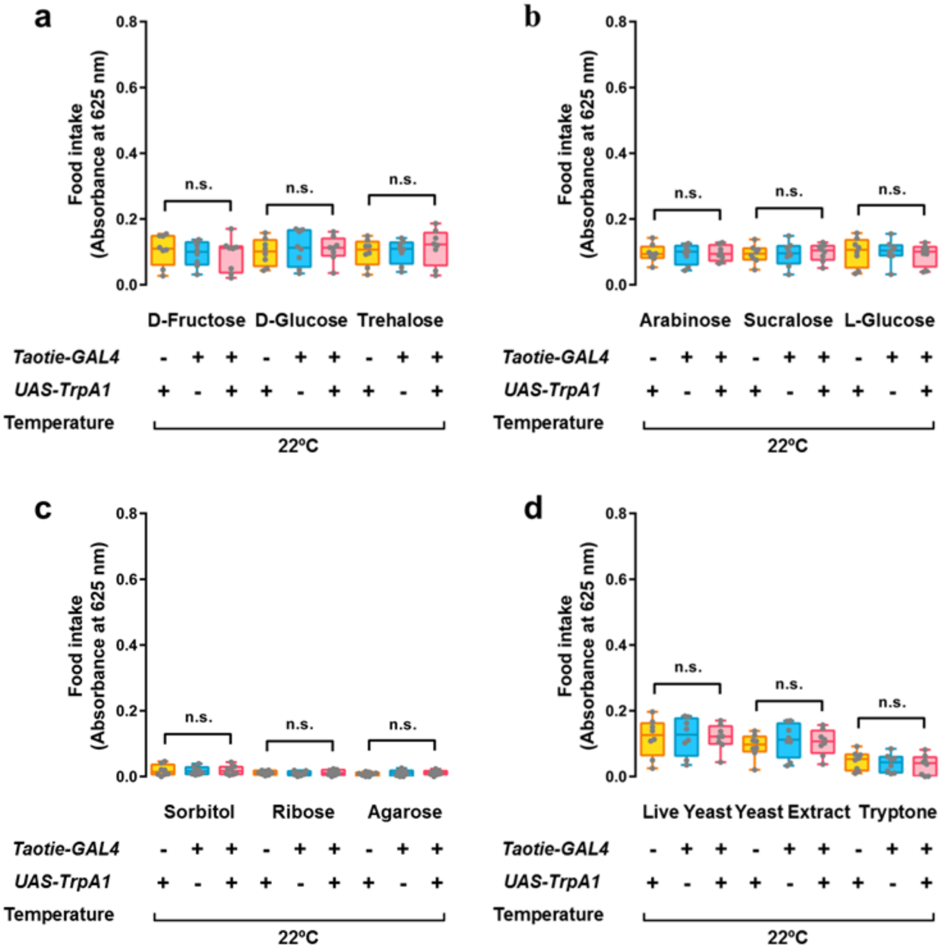

(a-d) Food intake of satiated *Taotie-GAL4*/*UAS-TrpA1* and genetic control flies on different carbohydrates or protein-rich diets at 22ºC (N = 8). All genotypes, temperatures, and experimental conditions are indicated with the plots. In a box and whisker plot, whiskers mark minimum and maximum, box includes 25th to 75th percentile, and the line in box indicates median of the data set. n.s. indicates not significant ($p > 0.05$) (ANOVA with Bonferroni post hoc test for multiple comparisons).

Supplementary Figure 5. Behavioral responses of activation of Taotie neurons in mixed-sugar experiments.

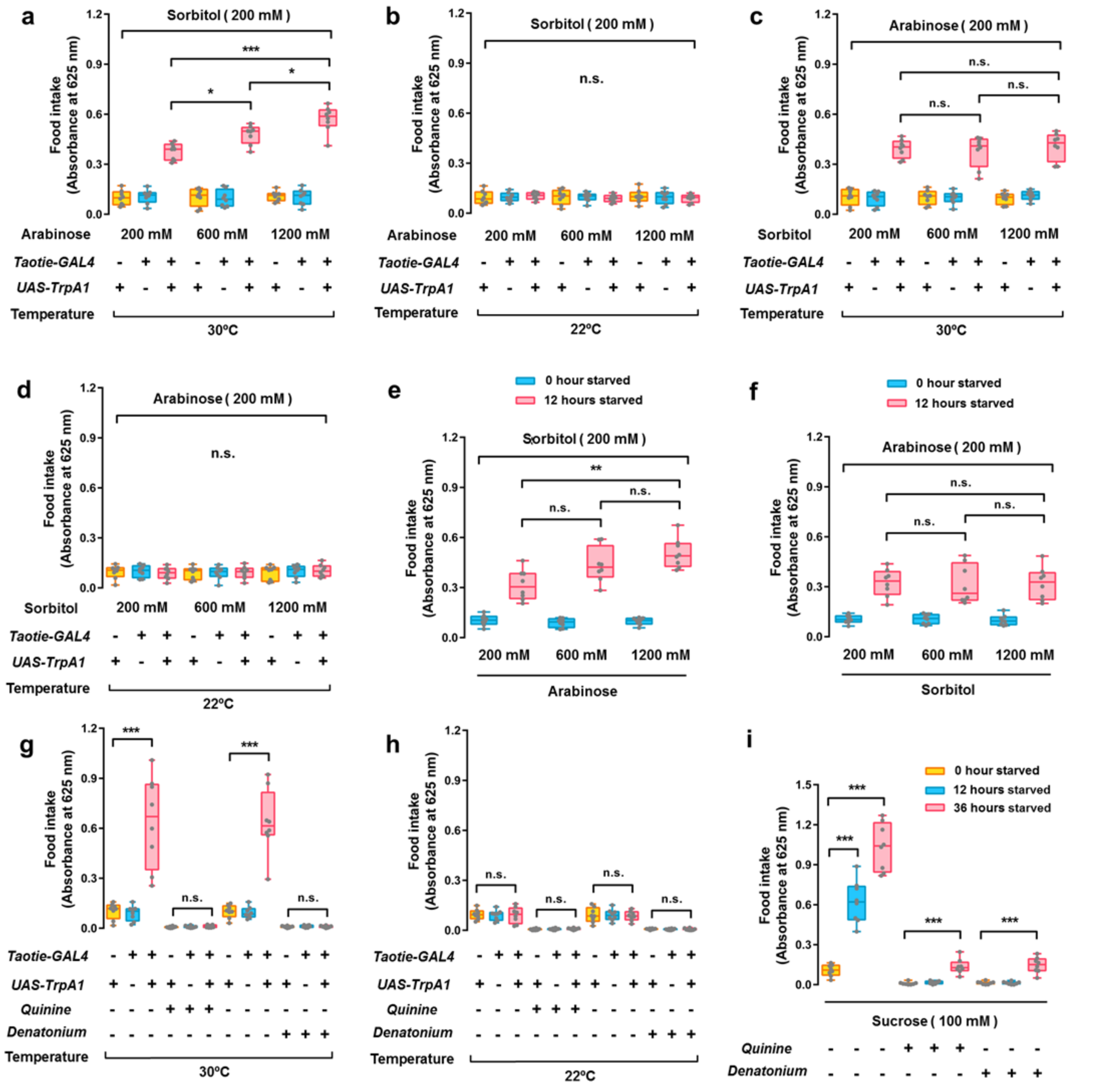

(a,b) Satiated *Taotie-GAL4*/*UAS-TrpA1* and genetic control flies tested with different concentrations of arabinose (sweet) added with tasteless but nutritious sorbitol (200 mM) at 30ºC and 22ºC (N = 8). (c,d) Satiated *Taotie-GAL4*/*UAS-TrpA1* and genetic control flies tested with different concentrations of sorbitol added with arabinose (200 mM) at 30ºC and 22ºC (N = 8). (e,f) Food consumption of wild type flies with sugar mixes after starvation for 12 hours (N = 8). (g,h) Food consumption of satiated

Taotie-GAL4/*UAS-TrpA1* and genetic control flies on sucrose mixed with quinine (1 mM) or denatonium (1 mM) at 30ºC and 22ºC (N = 8). (i) Food consumption of wild type flies starved for 12 and 36 hours toward sucrose mixed with quinine or denatonium. All genotypes, temperatures, and experimental conditions were as indicated with the plots. In a box and whisker plot, whiskers mark minimum and maximum, box includes 25th to 75th percentile, and the line in box indicates median of the data set. n.s. indicates not significant ($p > 0.05$); *: $p < 0.05$, **: $p < 0.01$, ***: $p < 0.001$ (Student's *t* test within each genotype for two group-only comparisons, ANOVA with Bonferroni post hoc test for multiple comparisons).

Supplementary Figure 6. Thermogenetic activation of Taotie neurons results in prolonged feeding motivation.

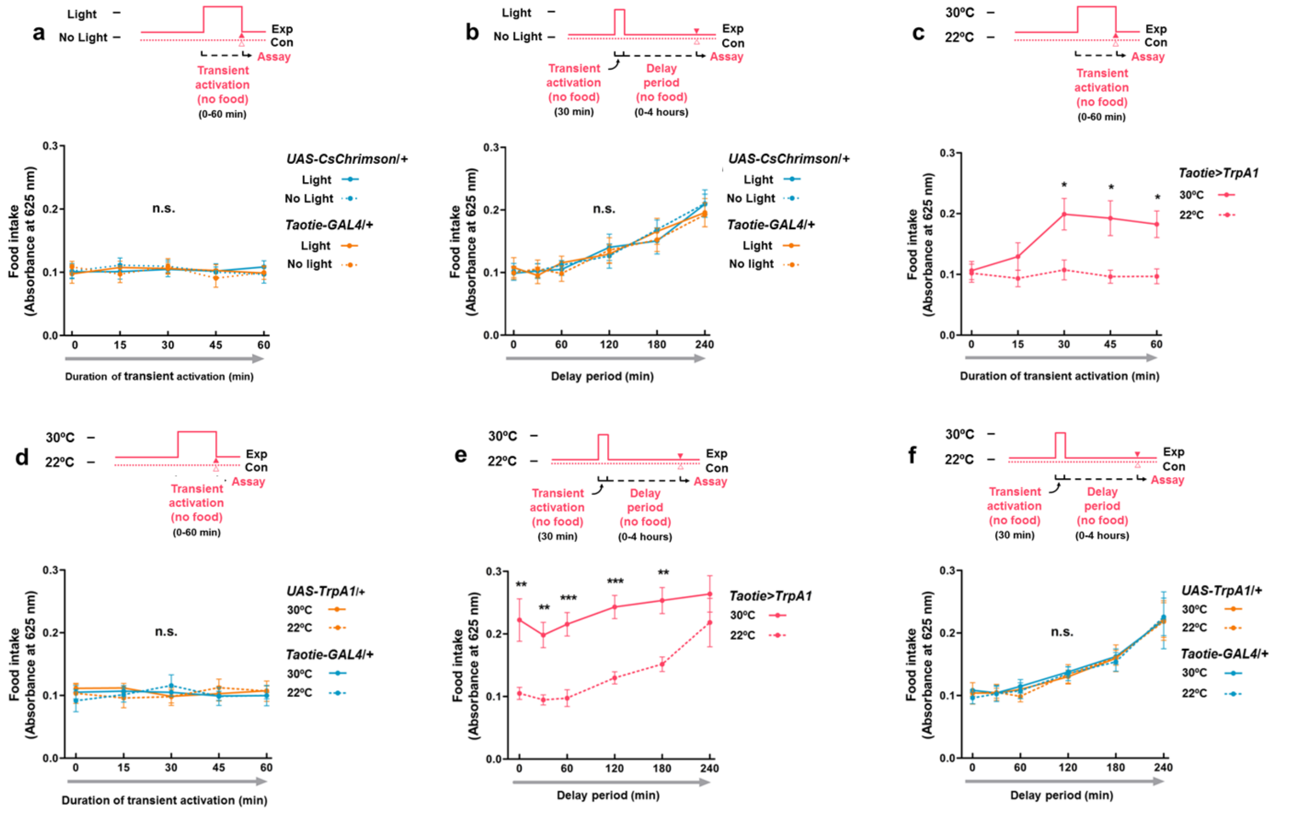

(a) Food intake of satiated *Taotie-GAL4*/+ and *UAS-Cschrimson*/+ flies. Flies were transiently activated by orange light for 0-60 minutes, placed into the dark immediately and tested for food intake (N = 8). "No light" flies were subjected to the same procedure without light stimulation. (b) Food intake of *Taotie-GAL4*/+ and *UAS-Cschrimson*/+ flies with various time intervals between activation and feeding test (N = 8). During the intervals, the flies were kept in the dark without food. "No light" flies were subjected to the same procedure, but without light stimulation. (c,d) Satiated flies, either *Taotie-GAL4*/*UAS-TrpA1* (c) or genetic control (d), were transiently activated at 30ºC for different time periods (0-60 minutes), followed by a cooling period of 5 minutes. Subsequently, these flies were transferred into a new vial to be subjected to a feeding test at 22ºC. Control flies were kept at 22ºC and tested for food intake without activation (N = 8). (e,f) After transient activation at 30ºC, *Taotie-GAL4*/*UAS-TrpA1* (e) and genetic control flies (f) were kept at 22ºC for different time intervals without food prior to the feeding test. Control flies were subjected to similar procedure, but without

activation (N = 8-9). All genotypes, temperatures, and experimental conditions are indicated with the plots. n.s. indicates not significant ($p > 0.05$); *: $p < 0.05$, **: $p < 0.01$, ***: $p < 0.001$ (Student's *t* test within each genotype for two group-only comparisons). Error bars indicate s.e.m.

Supplementary Figure 7. Feeding in delay period suppresses feeding motivation.

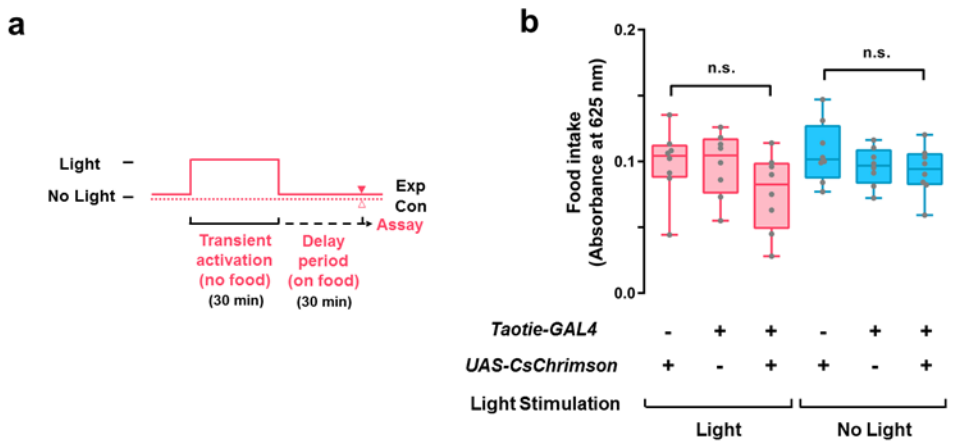

(a) Satiated flies of *Taotie-GAL4*/*UAS-CsChrimson* were transiently activated by orange light for 30 minutes, and then kept in the dark for 30 minutes with food, before being tested for food-intake. "No light" flies were subjected to the same procedure without light stimulation. (b) Food intake of *Taotie-GAL4*/*UAS-CsChrimson* after a 30-minute delay period with food (N = 8). During the delay interval, the flies were kept in the dark with food. All genotypes, temperatures, and experimental conditions were as indicated within the plots. In a box and whisker plot, whiskers mark minimum and maximum, box includes 25th to 75th percentile, and the line in box indicates median of the data set. n.s. indicates not significant ($p > 0.05$) (ANOVA with Bonferroni post hoc test for multiple comparisons).

Supplementary Figure 8. Overeating phenotype persists upon removal of Taotie neurons from the periphery.

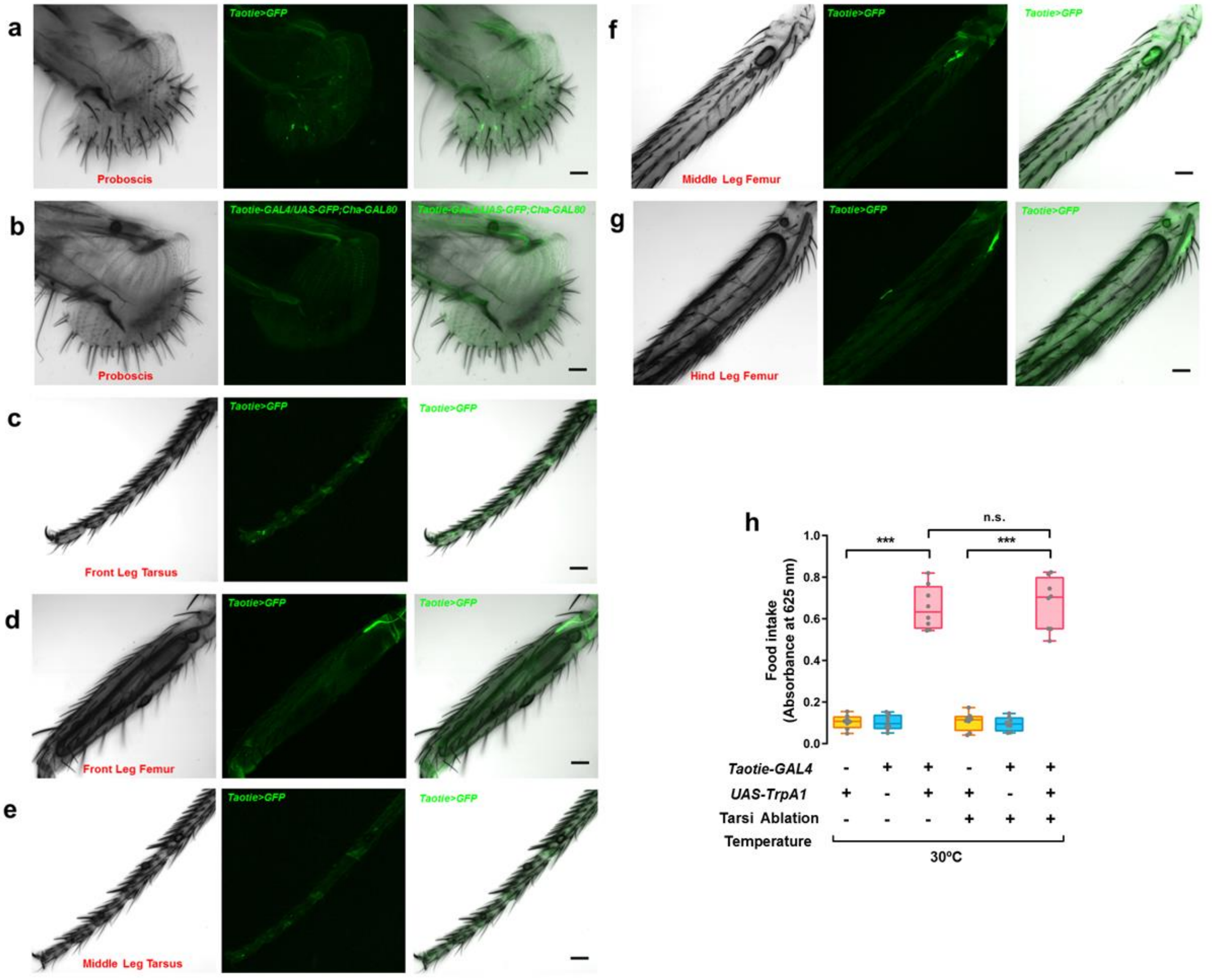

(a) Expression pattern of *Taotie-GAL4*/*UAS-mCD8-GFP* (green) in proboscis. (b) *Cha-GAL80* completely blocked *Taotie-GAL4* expression in proboscis. Scale bar, 50 µm. (c-g) Expression of *Taotie-GAL4*/*UAS-mCD8-GFP* (green) in front leg tarsus (c), front leg femur (d), middle leg tarsus (e), middle leg femur (f), and hind leg femur (g). Scale bar, 50 µm. (h) Food intake after tarsi ablation in *Taotie-GAL4*/*UAS-TrpA1* flies. The tarsi of front legs and middle legs were bilaterally removed by surgery. After 2-4 days of recovery, the feeding tests were conducted at 30ºC in satiated condition, with non-surgical flies as the controls (N = 8). All genotypes, temperatures, and experimental conditions were as indicated within the plots. In a box and whisker plot, whiskers mark minimum and maximum, box includes 25th to 75th percentile, and the line in box indicates median of the data set.

n.s. indicates not significant ($p > 0.05$); ***: $p < 0.001$ (ANOVA with Bonferroni post hoc test).

Supplementary Figure 9. Laser-targeted activation of Taotie neurons in different body regions.

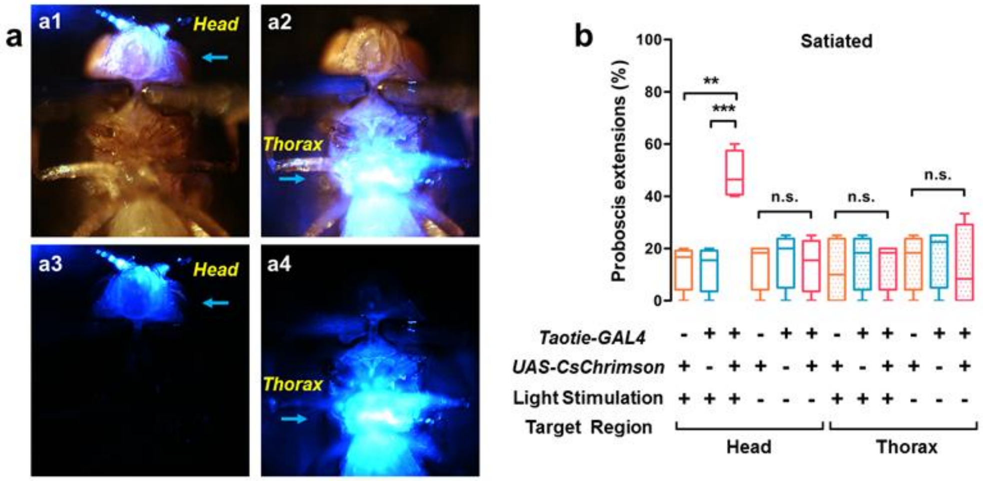

(a) Images showing the scale and location of laser spots on immobilized flies. a1 and a2 were taken with dim room light for visualization purpose. The location of head (a1, a3) and thorax (a2, a4) are indicated by blue arrows. (b) Fraction of satiated flies showing PER response following selective targeting of either head or thorax by a blue laser beam (N = 4, n = 4-7). All genotypes, temperatures, and experimental conditions were as indicated below the plots. In a box and whisker plot, whiskers mark minimum and maximum, box includes 25th to 75th percentile, and the line in box indicates median of the data set. n.s. indicates not significant ($p > 0.05$); **: $p < 0.01$, ***: $p < 0.001$ (ANOVA with Bonferroni post hoc test).

Supplementary Figure 10. Restricted expression patterns of Taotie neurons.

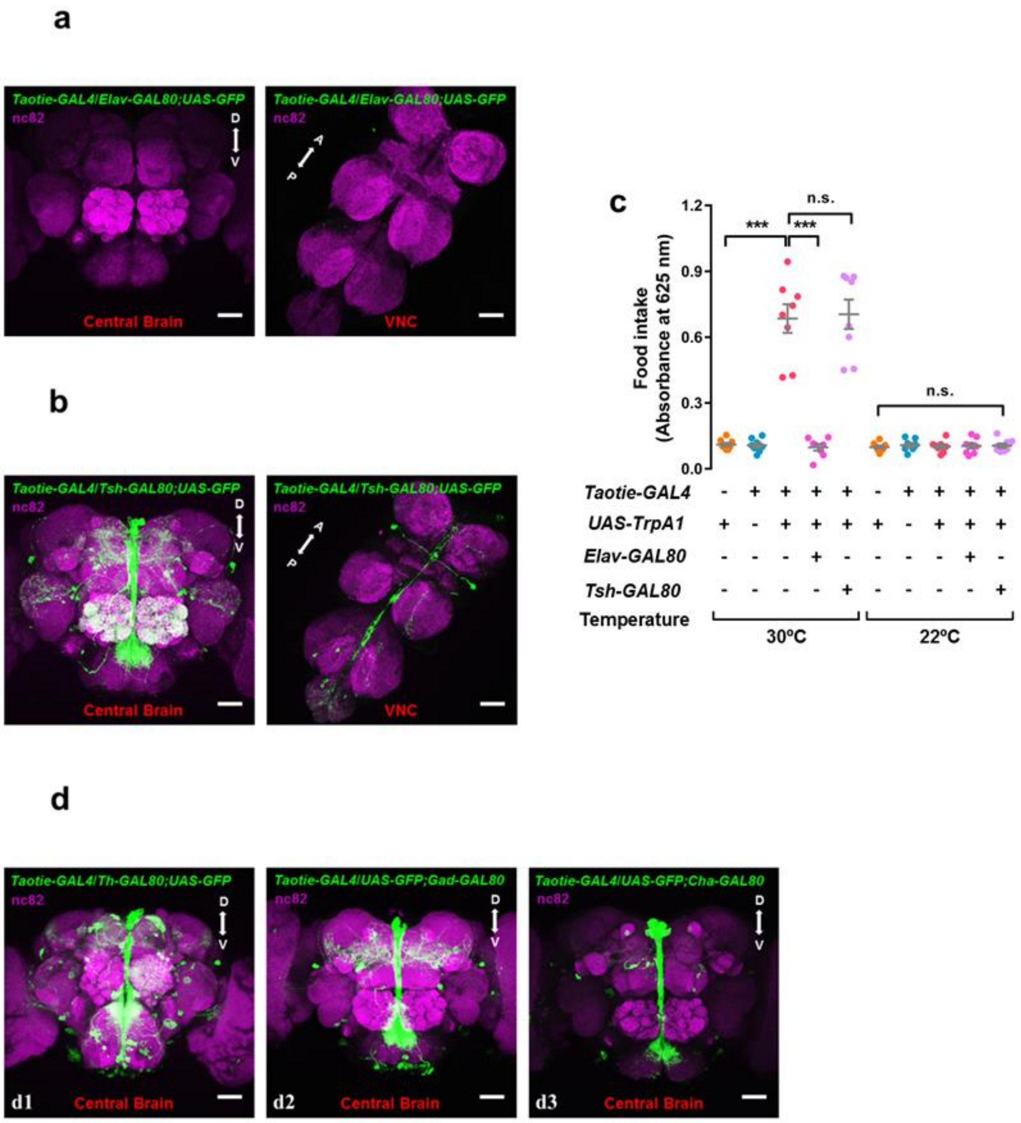

(a) Expression patterns of *Taotie-GAL4* in brain (left) and VNC (right) when combined with *Elav-GAL80*. (b) Expression patterns of *Taotie-GAL4* in brain (left) and VNC (right) when combined with *Tsh-GAL80*. (c) Food-intake of satiated *Taotie-GAL4/UAS-TrpA1* flies when containing either *Elav-GAL80* or *Tsh-GAL80* (N = 8). (d) Expression patterns of *Taotie-GAL4* in brain when combined with *Th-GAL80* (d1), *Gad-GAL80* (d2), and *Cha-GAL80* (d3). All brains were stained with a neuropil marker nc82 (magenta). Scale bar, 50 μm. All genotypes, temperatures, and experimental conditions are indicated with the plots. n.s. indicates not significant ($p > 0.05$); ***: $p < 0.001$ (Student's *t* test

within each genotype for two group-only comparisons). Error bars indicate s.e.m.

Supplementary Figure 11. Distribution of synaptic sites along Taotie neurons.

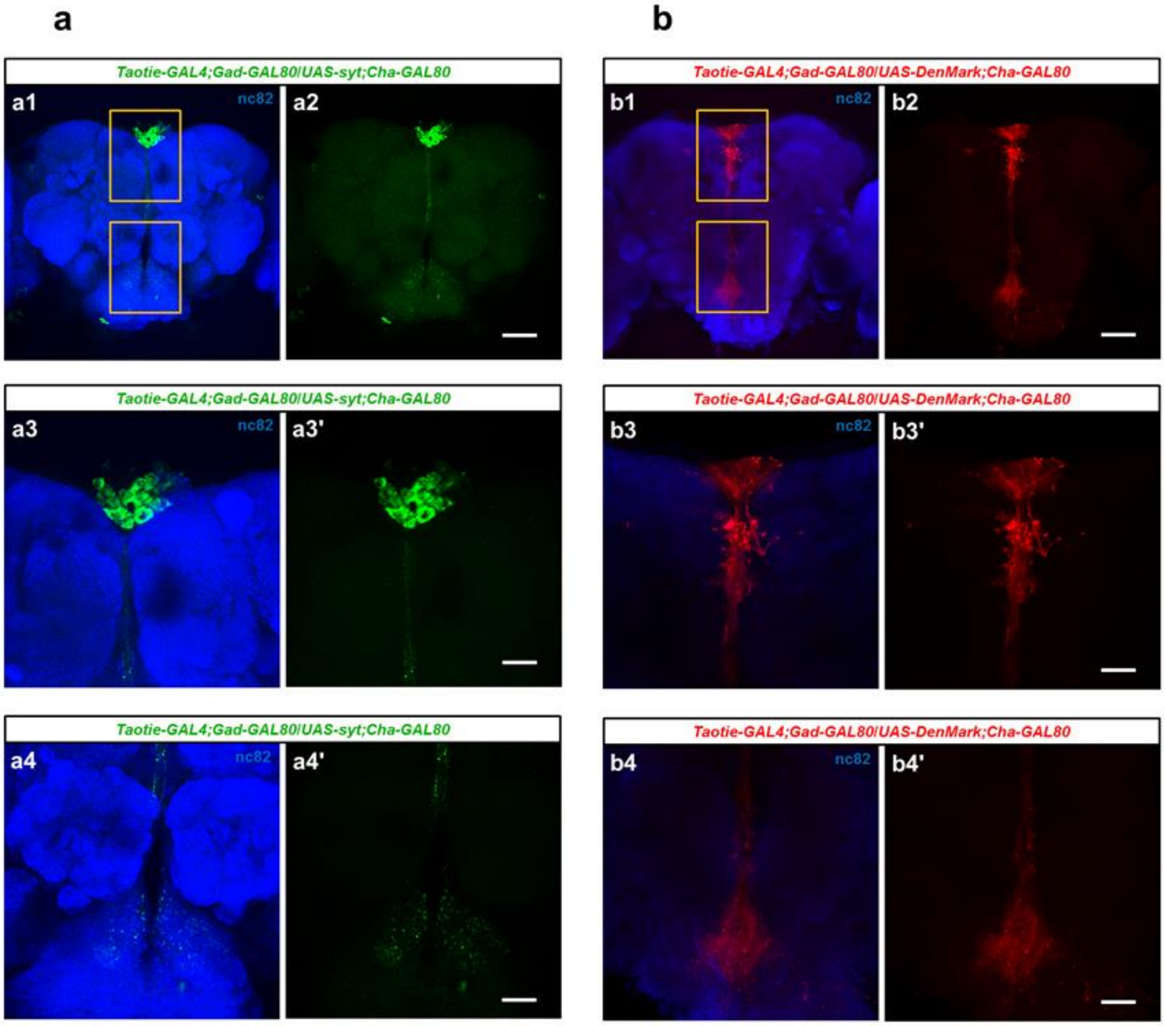

(a) *Taotie-GAL4* fly strain expressing the pre-synaptic marker *UAS-Syt-GFP.* a1 and a2: low magnification images showing the axonal projections of Taotie neurons. Scale bar, 50 µm. a3 and a3', a4 and a4': high magnification images of the boxed regions in a1 and a2. Scale bar, 20 µm. (b) *Taotie-GAL4* expressing the post-synaptic marker *UAS-DenMark.* b1 and b2: low magnification images showing the dendrites of Taotie neurons. Scale bar, 50 µm. b3 and b3', b4 and b4': high magnification images of the boxed regions in b1 and b2. Scale bar, 20 µm.

Supplementary Figure 12. Effects of Gr28b.b mutation and Gr28b.b overexpression on feeding behavior.

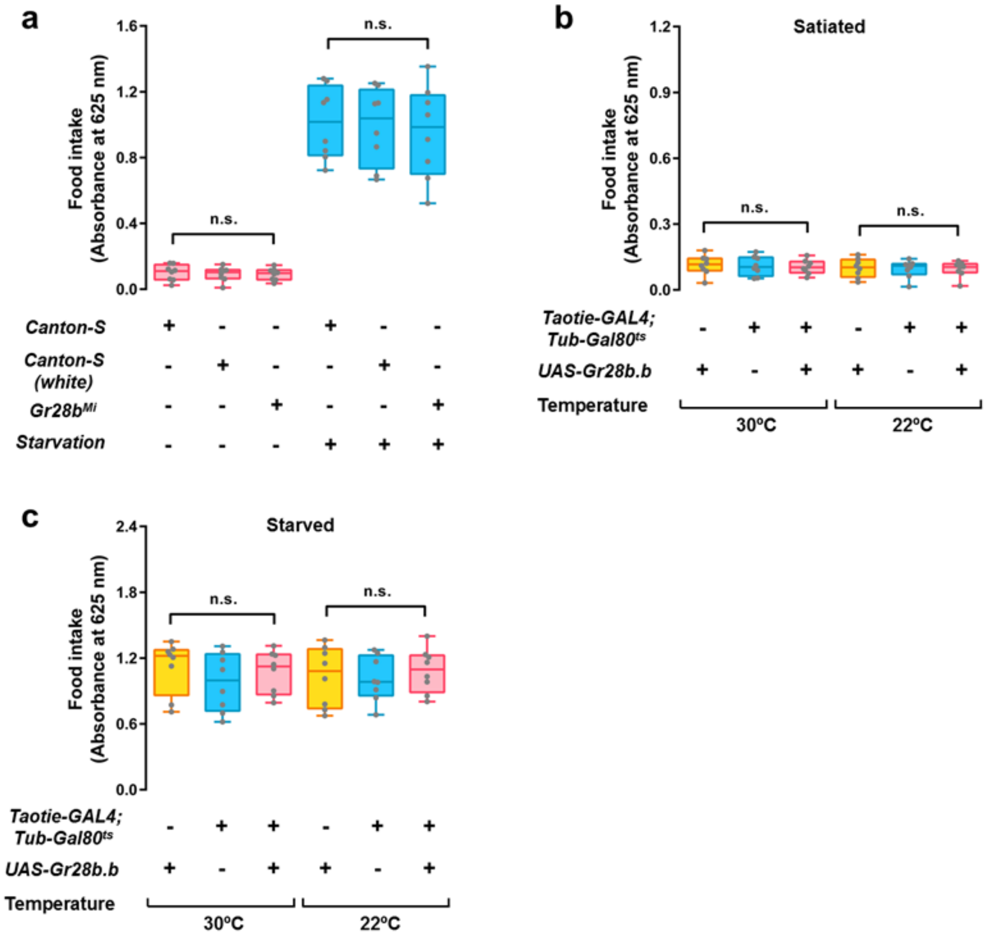

(a) Behavioral test of Gr28b mutants (Mi{ET1}Gr28bMB03888) under satiated and starvation conditions. *Canton-S* and *white* serve as controls (N = 8). (b,c) Food intake of flies with overexpression of *UAS-Gr28b.b* driven by *Taotie-GAL4;Tub-GAL80^{ts}* under satiated (b) and starved (c) conditions (N = 8). All genotypes, temperatures, and experimental conditions are indicated with the plots. In a box and whisker plot, whiskers mark minimum and maximum, box includes 25th to 75th percentile, and the line in box indicates median of the data set. n.s. indicates not significant ($p > 0.05$) (ANOVA with Bonferroni post hoc test for multiple comparisons).

Supplementary Figure 13. Decrease in feeding after interference with neuropeptide processing in Taotie neurons.

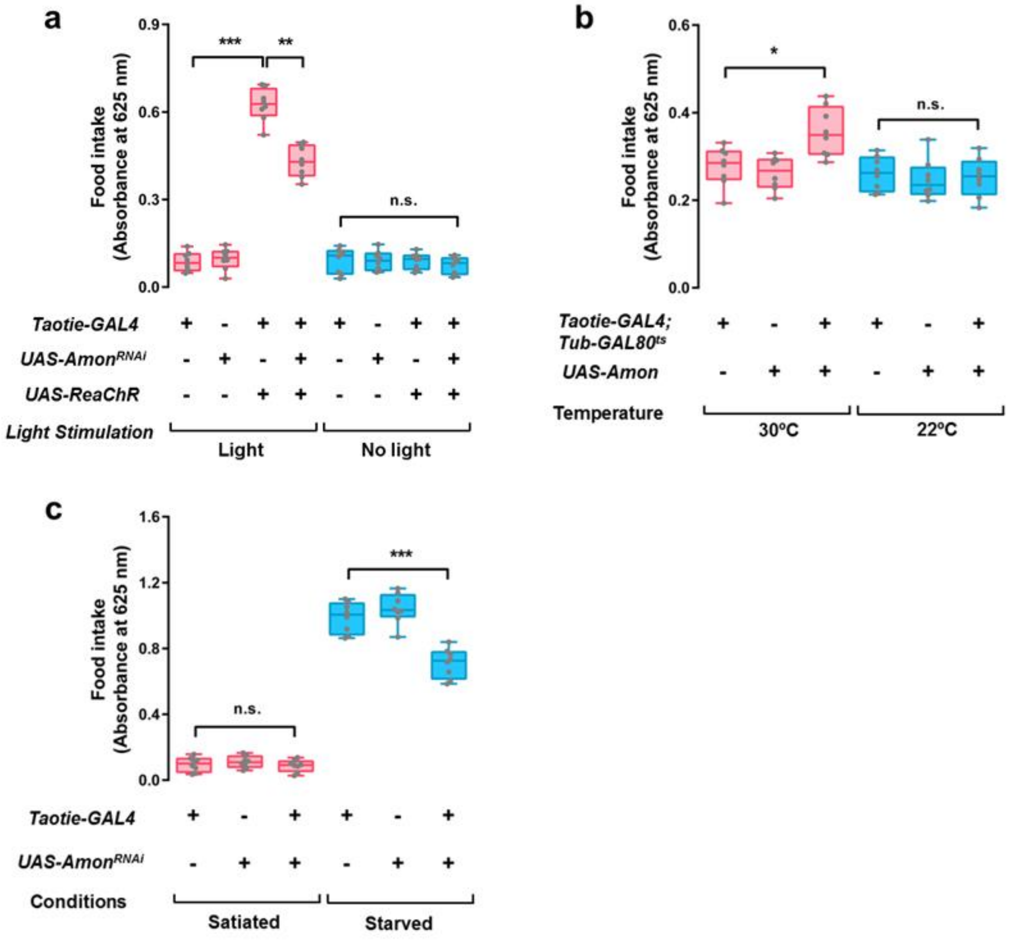

(a) Behavioral test in *Taotie>ReaChR* flies following the disruption of neuropeptide processing with *amon* RNAi (N = 8). (b) Food content after over-expressing *Amon* in Taotie neurons in adult flies. (N = 8). (c) Food consumption after knocking down *Amon* in Taotie neurons in satiated and starved flies. (N = 8). All genotypes, temperatures, and experimental conditions are indicated within the plots. In a box and whisker plot, whiskers mark minimum and maximum, box includes 25th to 75th percentile, and the line in box indicates median of the data set. n.s. indicates not significant ($p > 0.05$); *: $p < 0.05$, **: $p < 0.01$, ***: $p < 0.001$ (ANOVA with Bonferroni post hoc test for multiple comparisons).

Supplementary Figure 14. GCaMP signals and glucose levels in flies.

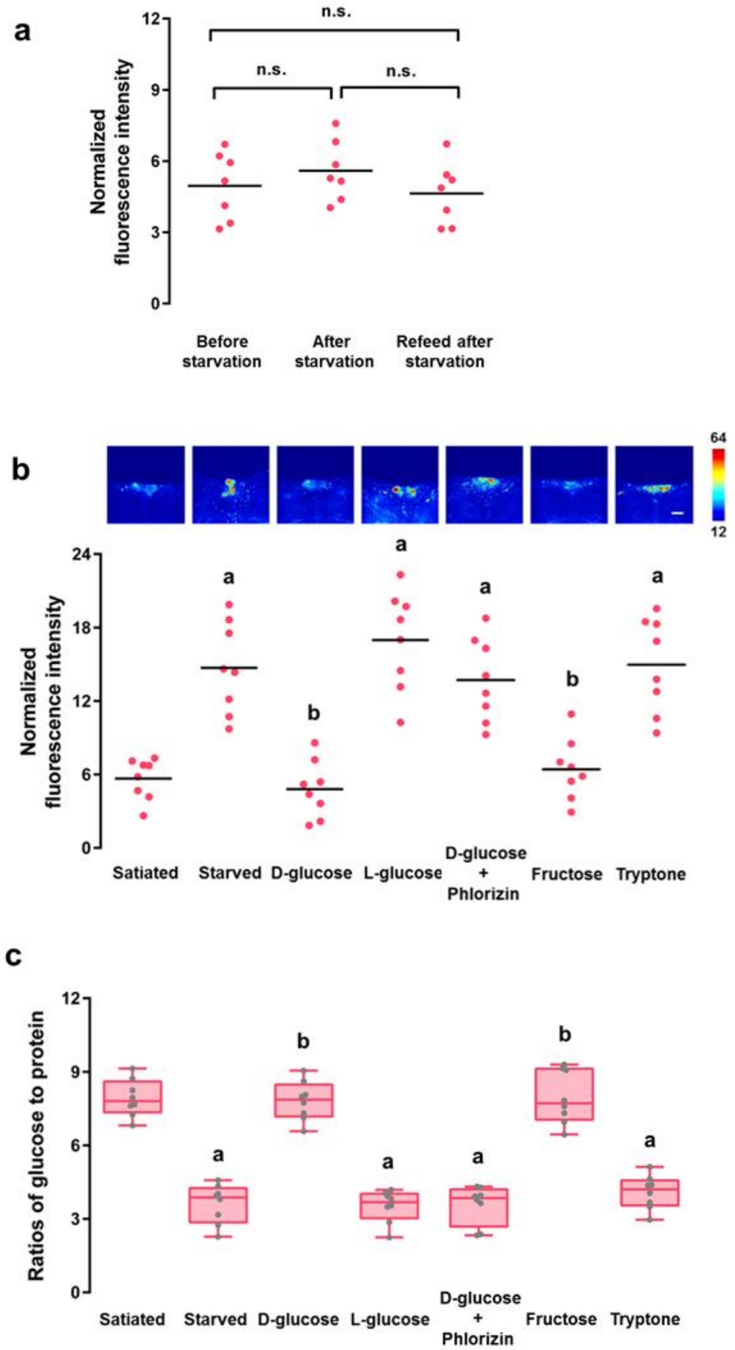

(a) No changes of calcium level were detected in the cells in the lateral protocerebrum. Comparison of GCaMP signals normalized to mCherry signals between groups of before-starvation, after-starvation and refeed-after-starvation (N = 7). Black bars indicate average signal intensity of each group. (b) Comparison of GCaMP signals normalized to RFP signals in *Taotie-GAL4/UAS-GCaMP6s;UAS-RFP*

flies re-feeding with different diets after 24 hours starvation (N = 8). Scale bar, 20 μm. (c) Glucose levels, normalized to protein levels, in *Taotie-GAL4*/*UAS-GCaMP6s;UAS-RFP* flies fed with different diets following 24 hours starvation (N = 8). All genotypes, temperatures, and experimental conditions are indicated within the plots. In a box and whisker plot, whiskers mark minimum and maximum, box includes 25th to 75th percentile, and the line in box indicates median of the data set. Different letters indicate groups with statistically significant differences. a: $p > 0.05$; b: $p < 0.001$ (Student *t* test within each genotype for two group-only comparisons).

Supplementary Figure 15. Comparison of the expression pattern of Taotie neurons with neurosecretory cells.

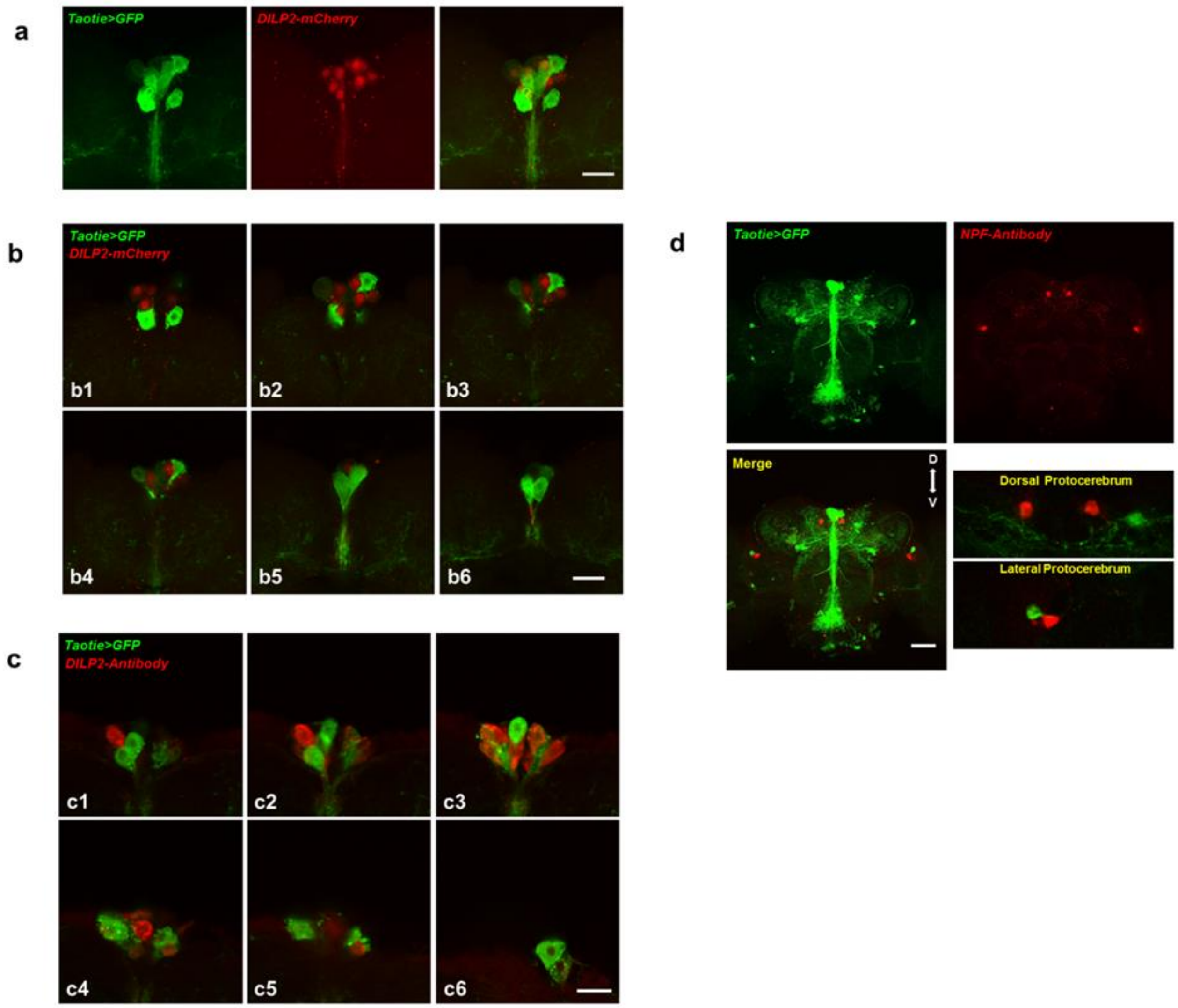

(a) Confocal images of the PI region in *Taotie-GAL4;UAS-mCD8-GFP/Dilp2-mCherry* flies. Scale bar, 15 μm. (b) A series of optical sections of the PI region in *Taotie-GAL4;UAS-mCD8-GFP/Dilp2-mCherry* flies (b1-b6). (c) Six continuous optical sections showing the PI region in *Taotie-GAL4/UAS-mCD8-GFP* flies immunostained with Dilp2-antibodies (c1-c6). Thickness of each section is 4 μm. Scale bar, 10 μm. (d) Images of the brain of T*aotie-GAL4/UAS-mCD8-GFP* (green) flies immunostained with anti-NPF antibody (red). Scale bar, 50 μm. No overlap was observed in the merged images (a-d). All genotypes were as indicated within

the plots.

Supplementary Figure 16. Activation of Dilp2 neurons reduces food intake.

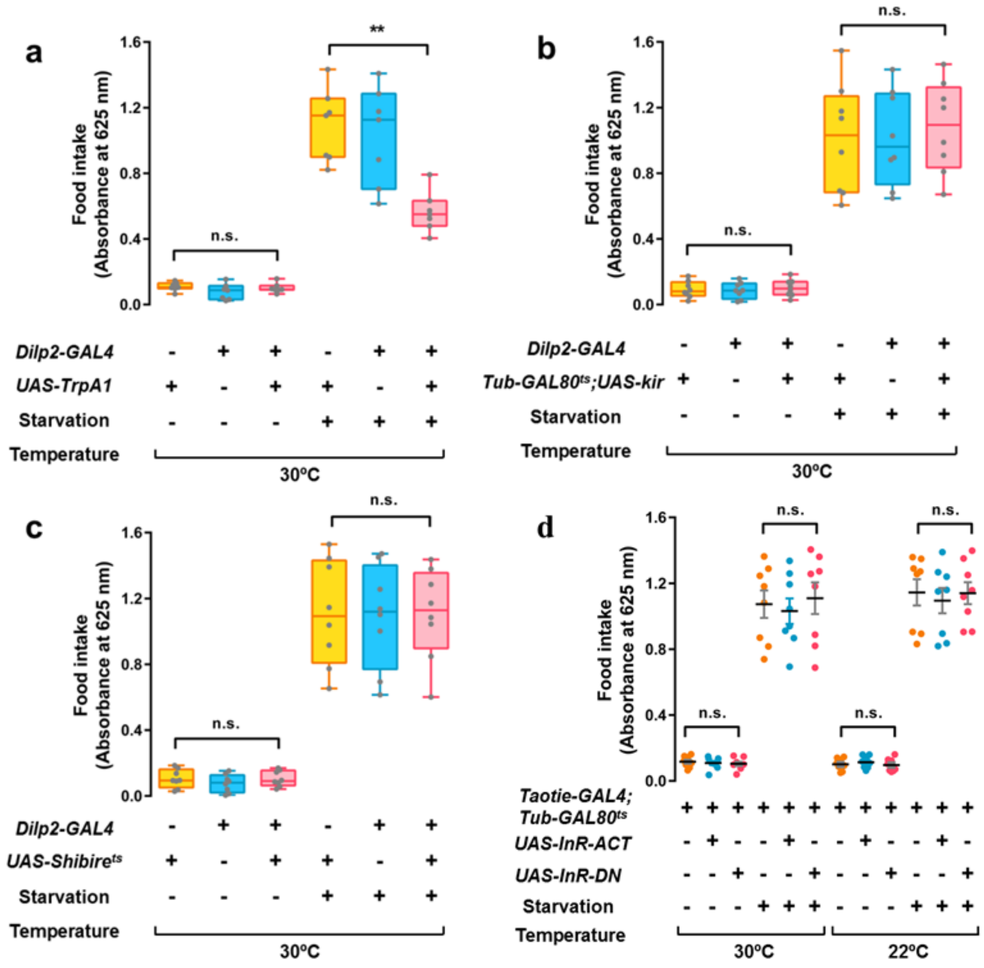

(a) The quantities of food ingested in *Dilp2>TrpA1* and control flies, under both satiated and starved conditions, in a feeding assay at 30ºC to activate Dilp2 neurons (N = 7-8). (b) Satiated and starved flies with *Dilp2-GAL4, Tub-GAL80*^{ts}, *UAS-Kir2.1* were tested for food-intake at 30ºC after blocking Dilp2 neurons (N = 8). All flies were reared at 22ºC. Prior the feeding tests, flies were subjected to a two-days treatment at 30ºC for heat-inducible expression of *Kir2.1*. (c) Food-intake of *Dilp2>Shibire*^{ts} and control flies after inactivation of Dilp2 neurons at 30ºC (N = 8). (d) Feeding of *Taotie-GAL4;Tub-GAL80*^{ts} flies with either *UAS-InR-ACT* (constitutively active) or *UAS-InR-DN* (dominant negative) under satiated and starved condition (N = 8). All genotypes, temperatures, and experimental conditions are indicated with the plots. In a box and whisker plot, whiskers mark minimum and maximum, box includes 25th to 75th percentile, and the line in box indicates median of

the data set. n.s. indicates not significant ($p > 0.05$); **: $p < 0.01$ (Student's *t* test within each genotype for two group-only comparisons). Error bars indicate s.e.m.

Supplementary Figure 17. Chronic activation of Taotie neurons elevates insulin signaling.

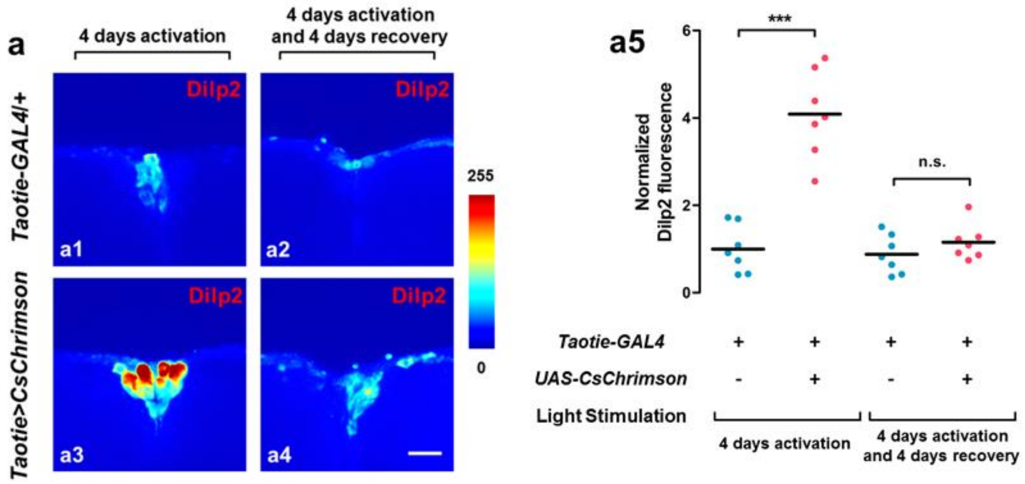

(a) The insulin signals in *Taotie-GAL4*/*UAS-CsChrimson* and genetic control flies after 4 days of continuous activation by light (a1, a3) or additional 4 days of recovery in darkness (a2, a4). Scale bar, 20 μm. (b) Quantifying insulin signals. All Dilp2 fluorescence intensities were normalized to that of *Taotie-GAL4*/+ flies with 4 days light activation (N = 7). All genotypes, temperatures, and experimental conditions are indicated within the plots. n.s. indicates not significant ($p > 0.05$); ***: $p < 0.001$ (Student's *t* test within each genotype for only two group-only comparisons).

Supplementary Figure 18. Taotie neurons and Dh44 neurons are distinct populations.

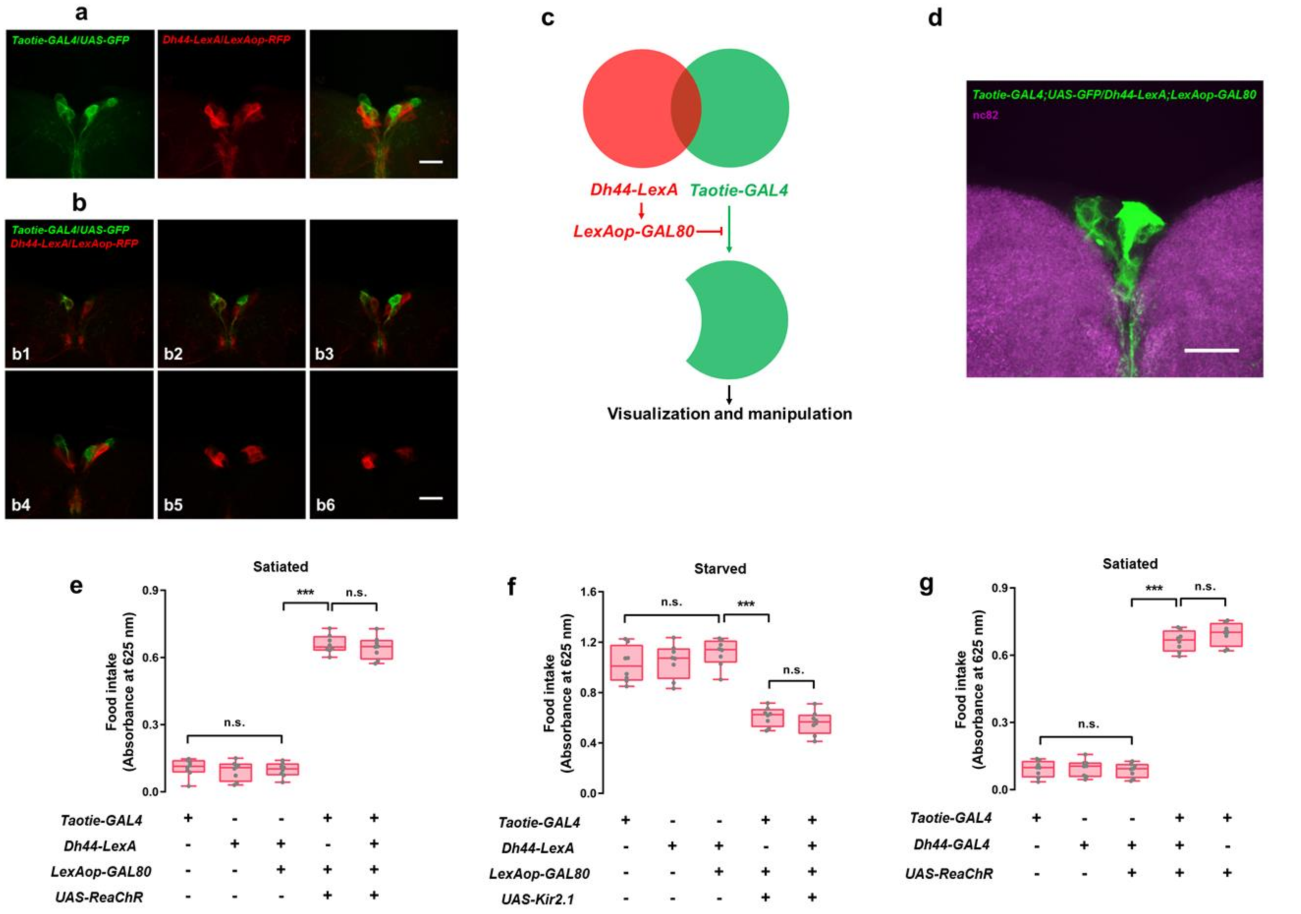

(a) A confocal stack of the PI region in *Taotie-GAL4;UAS-GFP*/*Dh44-LexA;LexAOP-mCD8-RFP* flies. Scale bar, 20 μm. (b) A series of optical sections of the PI region in *Taotie-GAL4;UAS-GFP*/*Dh44-LexA;LexAOP-mCD8-RFP* flies (b1-b6). Scale bar, 20 μm. (c) Strategy for intersection of Taotie neurons and Dh44 neurons in the PI region. The *UAS-effector* would be only expressed in the light green portion (the Taotie positive but Dh44 negative neurons). (d) Confocal images of the PI region in *Taotie-GAL4;UAS-mCD8-GFP*/*Dh44-LexA;LexAOP-GAL80* flies. Scale bar, 20 μm. (e) Food intake of satiated *Taotie-GAL4;UAS-ReaChR*/*Dh44-LexA;LexAOP-GAL80* and control flies after light stimulation. (f) Food intake of starved *Taotie-GAL4;UAS-Kir2.1*/*Dh44-LexA;LexAOP-GAL80* and control flies. (g) Food ingestion after co-activation of Taotie and Dh44 neurons in *Taotie-GAL4;UAS-ReaChR*/*Dh44-GAL4* flies. All genotypes, temperatures, and experimental conditions are indicated with the plots. In a box and

whisker plot, whiskers mark minimum and maximum, box includes 25th to 75th percentile, and the line in box indicates median of the data set. n.s. indicates not significant ($p > 0.05$); ***: $p < 0.001$ (Student *t* test within each genotype for two group-only comparisons, ANOVA with Bonferroni post hoc test for multiple comparisons).

Supplementary Figure 19. Epistatic interactions of Taotie neurons with known satiated signals.

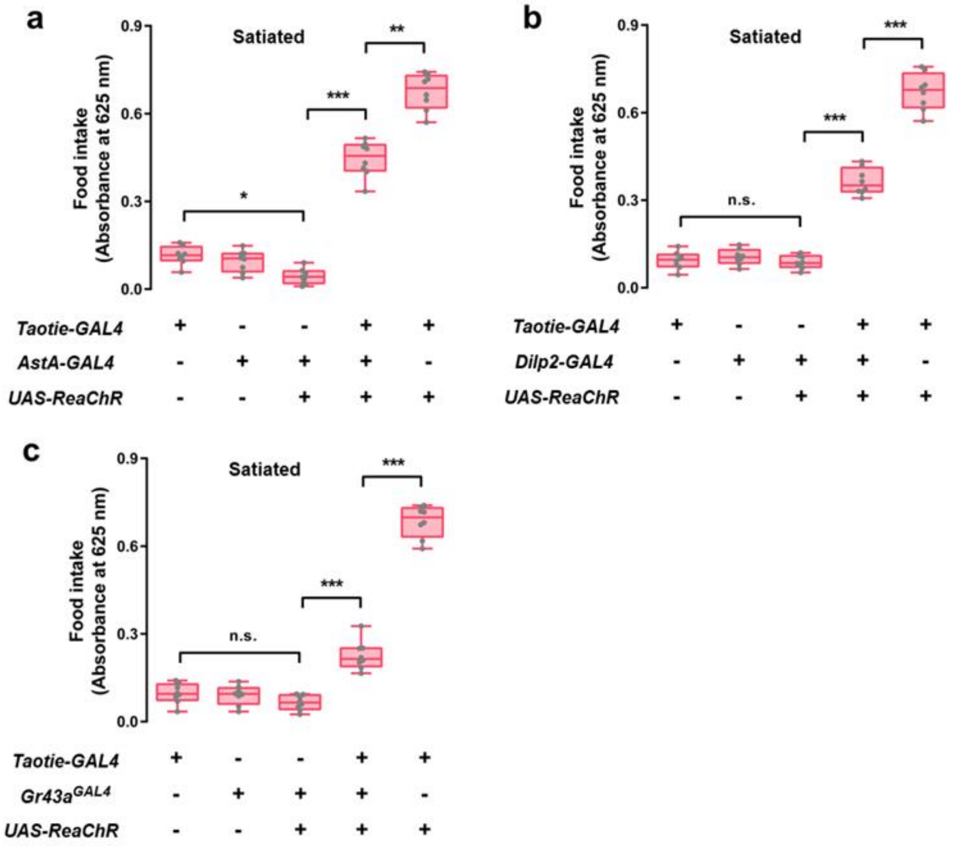

(a) Food intake after co-activation of Taotie and AstA neurons with *UAS-ReaChR* (N = 8). (b) Food intake after co-activation of Taotie and Dilp2 cells with *UAS-ReaChR* (N = 8). (c) Food intake after co-activation of Taotie and Gr43a neurons with *UAS-ReaChR* (N = 8). All genotypes, temperatures, and experimental conditions are indicated with the plots. In a box and whisker plot, whiskers mark minimum and maximum, box includes 25th to 75th percentile, and the line in box indicates median of the data set. n.s. indicates not significant ($p > 0.05$); *: $p < 0.05$, **: $p < 0.01$, ***: $p < 0.001$ (Student *t* test within each genotype for two group-only comparisons, ANOVA with Bonferroni post hoc test for multiple comparisons).

Supplementary Figure 20. Distribution of neurons related to feeding and metabolism in Drosophila.

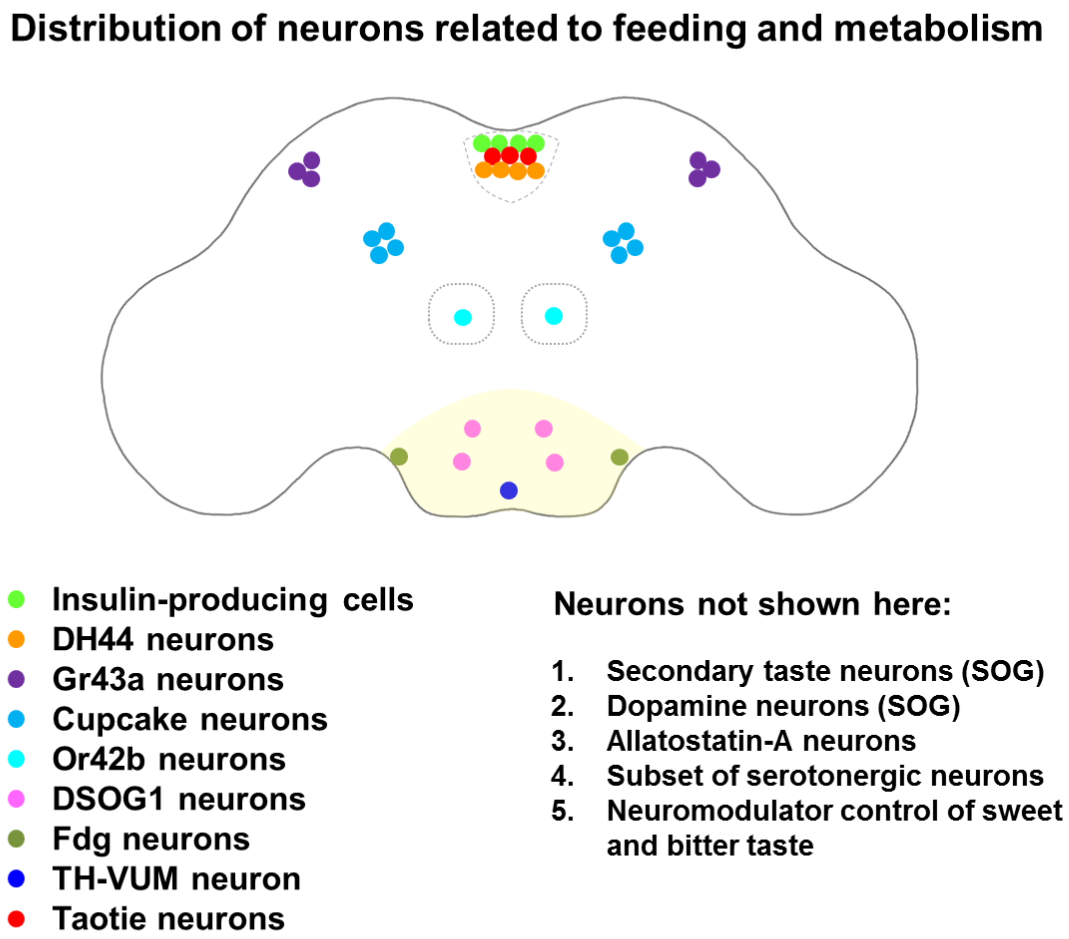

A diagram shown that Taotie neurons are anatomically distinct from previously identified neuromodulators involved in feeding behavior and energy balance.

Supplementary Table 1. The qPCR primers used in this study.

Rp49:	F: 5′-GCTAAGCTGTCGCACAAATG-3′;<br>R: 5′-GTTCGATCCGTAACCGATGT-3′;
Dilp2:	F: 5′-ATCCCGTGATTCCACACAAG-3′;<br>R: 5′-GCGGTTCCGATATCGAGTTA-3′;
Dilp3:	F: 5′-CAACGCAATGACCAAGAGAA-3′;<br>R: 5′-TGAGCATCTGAACCGAACT-3′;
Dilp5:	F: 5′-GCCTTGATGGACATGCTGA-3′;<br>R: 5′-AGCTATCCAAATCCGCCA-3′;
Eiger:	F: 5′-GATGGTCTGGATTCCATTGC-3′;<br>R: 5′-TAGTCTGCGCCAACATCATC-3′;
Upd2:	F: 5′-CGGAACATCACGATGAGCGAAT-3′;<br>R: 5′-TCGGCAGGAACTTGTACTCG-3′;
Upd3:	F: 5′-ACTGGGAGAACACCTGCAAT-3′;<br>R: 5′-GCCCGTTTGGTTCTGTAGAT-3′;
Domeless:	F:5′-ATCGCAAAGAATACAAAATAAATTACAAAC-3′;<br>R: 5′-TCTGGAATCTGGAAACTAGAAACCAC-3′;
InR:	F: 5′-TCTCAGTCATGATGATATC-3′;<br>R: 5′-GTGACAGTTGCTAAAGAT-3′;
Ds6k:	F: 5′-AGGATGCGGCGGCTGTTC-3′;<br>R: 5′-CATCAGGTGAATCCACTG-3′;
4E-BP:	F: 5′-CATGCAGCAACTGCCAAATC-3′;<br>R: 5′-CCGAGAGAACAAACAAGGTGG-3′;
eIF4E:	F: 5′-GACTTCTGGAGCCTATAC-3′;<br>R: 5′-CAGGCAGAGCAGCACATC-3′;
